# Teamworking in Healthcare during the COVID-19 Pandemic: A Mixed-Method Study

**DOI:** 10.3390/ijerph181910371

**Published:** 2021-10-01

**Authors:** Sabrina Anjara, Robert Fox, Lisa Rogers, Aoife De Brún, Eilish McAuliffe

**Affiliations:** UCD Centre for Interdisciplinary Research, Education, and Innovation in Health Systems (UCD IRIS), Health Sciences Centre, School of Nursing, Midwifery & Health Systems, University College Dublin, D04 V1W8 Dublin, Ireland; sabrina.anjara@gmail.com (S.A.); robert.fox@ucd.ie (R.F.); lisa.rogers@ucd.ie (L.R.); eilish.mcauliffe@ucd.ie (E.M.)

**Keywords:** collective leadership, COVID-19, teamwork, psychological safety, work engagement, organisational citizenship, mixed methods

## Abstract

The widespread impact of COVID-19 on healthcare has demanded new ways of working across many organisation types and many forms of healthcare delivery while at the same time endeavouring to place minimal, or no, additional burden on already strained healthcare teams. This is a cross-sectional mixed-method study which captured the experiences of teamwork during the COVID-19 pandemic contributing to successful collaboration. We hypothesised that work engagement and psychological safety separately contribute to collective leadership and organisational citizenship behaviours. Participants were healthcare staff on active duty during the COVID-19 pandemic in Ireland (*n* = 152) who responded to our social media (Twitter) invitation to participate in this study. Survey and free-text responses were collected through an online platform. Structural equation modelling examined the relationships between work engagement and psychological safety, and collective leadership and OCBs. Open text responses relating to experiences of teamworking during the pandemic were analysed for latent themes. From the survey data, the structural model demonstrated excellent statistical fit indicating that psychological safety, but not work engagement, was predictive of collective leadership and OCBs. From the qualitative data, two key themes were generated: (1) Contrasting experiences of working in a team during the pandemic; and (2) The pandemic response: a tipping point for burnout. This study offers a valuable starting point to explore the factors driving change and the shift to more collective ways of working observed in response to COVID-19. Future studies should use longitudinal data to capture the temporal relationship of these variables which could be moderated by prolonged pressure to healthcare staff during the pandemic.

## 1. Introduction

The COVID-19 pandemic elicited an extraordinary response from healthcare teams. To reduce the transmission of the virus and to ensure the safe continuity of services, changes were rapidly implemented across all levels of health systems globally [1]. Despite the typically slow pace of change in healthcare pre-pandemic [2,3], new initiatives have been rapidly approved and implemented. The widespread impact of COVID-19 on healthcare has demanded quicker designing, implementing, and learning about innovations across many organisation types and many forms of healthcare delivery while at the same time endeavouring to place minimal, or no, additional burden on already strained healthcare staff [4]. Whilst there is considerable variety of new initiatives introduced during the pandemic, such as the use of tablets to allow the family of patients in critical care to see their loved ones, common characteristics can be identified including the emergence of enhanced interprofessional collaboration and trust within healthcare teams and empowerment and autonomy to cultivate change [5]. Against the backdrop of intense pressure to maintain quality standards while keeping themselves safe, how did healthcare staff experience teamwork during the pandemic response? What were the factors that supported healthcare teams to deliver rapid changes in service delivery? This cross-sectional study explored the association between collective leadership, psychological safety, work engagement, and organisational citizenship behaviours during this time of exceptional transformation in healthcare.

### 1.1. Theoretical Approaches

During the pandemic, researchers have reported softer hierarchies and greater staff autonomy within multidisciplinary teams MDTs; [6,7]. This approach to patient care aligns with a shift away from traditional ‘command and control’ leadership styles to a more collaborative and collective leadership approach. While there have been many studies on traditional, formal leadership roles and how they influence the team environment, there is a paucity of research looking into collective leadership. Collective leadership is characterised by all team members jointly participating in decision-making processes and fulfilling tasks traditionally reserved for a hierarchical leader [8]. In the understanding of collective leadership, even those without formal leadership roles could contribute to the team’s decision-making processes. This differs from teamwork, which is generally understood as the activity of working together in a group with other people. One can have teamwork despite being in a team controlled by an authoritative leader.

Approaches such as collective leadership (e.g., shared or distributed leadership) emphasise the relational aspects of leadership, conceptualising leadership as a dynamic, interactive group-level phenomenon rather than the responsibility of one formal ‘heroic’ leader [9]. A recent meta-analysis by Wu et al. [8] has shown the positive relationship between shared leadership and group behaviour processes (e.g., problem solving), attitudinal outcomes (e.g., team satisfaction), team cognition (e.g., team creativity), and team performance (e.g., team productivity). Although research in other fields has begun to investigate the antecedents that support the emergence of collective leadership [10,11,12], our understanding of the factors that positively influence and enable collective leadership in healthcare teams is still developing. A recent systematic review of the literature found that internal team environment (i.e., shared purpose, voice and social support) and team heterogeneity are antecedents that are positively related to the emergence of shared approaches to leadership [8] Building on Carson et al.’s [10] work, we predicted that the internal team environment will support the emergence of collective leadership in healthcare teams.

One of the key characteristics of a positive team environment is one that promotes psychological safety [10]. To actively participate in patient care decision-making, staff must perceive that their work setting accepts and encourages collaboration and feedback [13]. Psychological safety refers to the shared belief that a work setting is a safe place to take interpersonal risks such as speaking up, asking questions, and sharing ideas and opinions [14]. The importance of psychological safety in healthcare teams is emphasised by the ongoing, global response to COVID-19; the continuous adaptation and redesign of services has required enhanced collaboration, engagement, creativity, innovation, and knowledge sharing across teams and across organisations. Newman et al. [15] identify these factors as key outcomes observed when working within psychologically safe environments. Previous research suggests that psychological safety enhances the adoption of leadership roles within MDTs by enabling involvement and voice in decision-making [16]. We acknowledge that it is also plausible that collective leadership would contribute to psychological safety within the team.

For healthcare staff to adopt leadership roles, they must be engaged and motivated to do so. Researchers conceptualise work engagement as a cognitive state in which individuals invest their personal resources and energies into their work roles and tasks [17,18]. Schaufeli et al. [19] consider vigour (e.g., high levels of energy and mental resilience while working), dedication (e.g., sense of enthusiasm or pride in one’s work), and absorption (e.g., being deeply engrossed in one’s work) as key characteristics of work engagement. The extant literature suggests that engagement is essential for overcoming the complex barriers associated with healthcare provision, for instance excessive workloads and inadequate staffing levels, and enables employees to feel attachment and engagement to their work roles [13,17,20]. When a psychologically safe environment exists, employees perceive greater self-determination and interest in their work, leading to improved innovation and shared learning [21]. Bakker and Albrecht [22] also suggest that because engaged employees are open to new experiences, staff are more inclined to help their colleagues. Similarly, Kahn [17] suggests that engaged individuals are more likely to step outside the formal boundaries of their role to assist their colleagues and support the goals of their team or organisation. Although research has examined the association between transformational leadership (which focuses on the behaviour of a designated leader) and work engagement [23,24], the relationship between work engagement, collective leadership and extra-role behaviours remains unclear, requiring further investigation.

Organisational citizenship behaviour (OCB) is a term used to describe these extra-role behaviours which include helping colleagues, encouraging others, and volunteering to take on additional responsibilities [25,26]. In addition to work engagement and collective leadership, psychological safety has also been positively associated with OCBs [20]. When healthcare staff feel comfortable taking interpersonal risks, they actively engage as part of the MDT and therefore we propose that staff may subsequently participate in extra-role behaviours to support their colleagues.

### 1.2. Hypotheses

This study aims to capture the experiences of teamwork during the COVID-19 pandemic, exploring factors which contribute to successful collaboration. We hypothesise that psychological safety is a pre-requisite conditions that promote collective leadership and OCBs in healthcare teams. Acknowledging that psychological safety can also contribute to work engagement [21], we additionally hypothesise that work engagement (even in the absence of psychological safety) promotes collective leadership and OCBs in healthcare teams during the pandemic, given the needs of the health service. As of the writing of this paper, no peer-reviewed studies have examined the four constructs together in the context of healthcare, especially with the lens of teamworking during the COVID-19 pandemic.

## 2. Materials and Methods

### 2.1. Design, Participants and Recruitment Strategy

This is a cross-sectional mixed-method study. Given the growing complexity of healthcare, we hoped to capture the richness of contextual perspectives and relationships that exist between the four constructs (psychological safety, work engagement, collective leadership, OCBs) beyond numerical data. To capture the corresponding circumstances of collaboration, we added open-ended questions to the survey. To ensure neutrality and provide a safe space for participants, we recruited through social media rather than healthcare institutions.

Participants (*n* = 152) in this study consisted of healthcare professionals who were working during the COVID-19 pandemic (from March 2020) in Ireland. They included clinical, administrative, and support staff. Participants were recruited using an online survey, completed via Qualtrics.com. The survey link was published on Twitter once a week during the study period, starting on 7 September 2020, and re-tweeted 230 times by Twitter users. Participants accessed the online survey using their own personal computer (desktop/laptop/tablet) or smartphone. Those clicking on the link were taken to an information and consent page. The survey was conducted entirely online with a median completion time of 15 min. This study received an exemption from full ethical review due to the low-risk nature of the work, from the research ethics committee at University College Dublin, Ireland.

### 2.2. Materials

This research employed four standardised scales and a series of open text questions relating to participants’ experiences of teamworking in healthcare during COVID-19. In addition, there was an optional section on demographic information. As Ireland has a relatively small population size and considering the self-reporting of racial data, to protect the anonymity of participants, age was reported in bands.

#### 2.2.1. Collective Leadership

Collective leadership was assessed using the Collective Leadership Scale [27]. This is a 25-item instrument which assesses four domains of collective leadership: planning and organizing (six items); problem solving (seven items); support and consideration (six items); and development and mentoring (six items). However, given the considerable time pressures on healthcare staff and to reduce participant response burden, only the first three domains were retained, totalling 19 items. The development and mentoring subscale had items pertaining to skills acquisition and exchanging career-related advice, which the researchers were aware would be difficult to do as non-essential in-person interactions were severely limited in the early stages of pandemic response. To avoid contaminating the integrity of the responses, we omitted this subscale. Items prompted participants to consider how often their team shared in tasks including ‘Planning how the work gets done’ and ‘Finding solutions to problems affecting team performance’. All items were rated using a seven-point Likert scale (‘rarely’ = 0, ‘always’ = 7), with higher scores reflecting higher levels of collective leadership. The psychometric properties of this measure have previously been supported [27]. Similarly, the internal consistency (Cronbach’s alpha) for the full scale (α = 0.98) and each individual subscale (planning and organising = 0.95; problem solving = 0.95; support and consideration = 0.95) in the current sample demonstrated high levels of consistency.

#### 2.2.2. Utrecht Work Engagement

Work engagement was measured using the Utrecht Work Engagement Scale UWES [19]. The UWES is comprised of 17 items that measure three dimensions of work engagement: vigour (six items), dedication (five items), and absorption (six items). Sample items include ‘When I get up in the morning, I feel like going to work’ and ‘At my job, I always persevere, even when things do not go well’. All items were rated using a seven-point Likert scale (‘rarely’ = 0, ‘always’ = 7), with higher scores reflecting increased work engagement. The psychometric properties of this measure have previously been supported [28]. Moreover, the internal consistency of the full scale (α = 0.95) and individual subscales (vigour = 0.90; dedication = 0.90; absorption = 0.83) were excellent in the current sample.

#### 2.2.3. Organisational Citizenship Behaviour

OCB was measured using the Organisational Citizenship Behaviour Scale [29]. This is a 24-item scale which assesses five domains of organisational citizenship behaviour: altruism; conscientiousness; sportsmanship; courtesy; and civic virtue. However, to minimise participant response burden and avoid overlap with UWES, only three domains were retained, namely altruism (five items), courtesy (five items), and civic virtue (four items), totalling 14 items. Items were rated using a seven-point Likert scale (‘strongly disagree’ = 0, ‘strongly agree’ = 7), with higher scores reflecting higher levels of OCB. Sample items on this scale prompted participants to consider whether team members ‘are mindful of how their behaviour affects other people’s jobs’ and ‘keep abreast of changes in the organisation’. The psychometric properties of this measure have previously been supported [29]. Moreover, the internal consistency of the full scale (α = 0.96) and individual subscales (altruism = 0.83; courtesy = 0.95; civic virtue = 0.95) were satisfactory in the current sample.

#### 2.2.4. Psychological Safety

Psychological safety was assessed using the 19-item Psychological Safety Scale [30]. This recently developed measure is designed to target psychological safety among healthcare professionals in relation to their team leader (nine items), fellow team members (seven items), and the whole team (three items). All items were rated using a seven-point Likert scale (‘strongly disagree’ = 0, ‘strongly agree’ = 7), with higher scores being indicative of higher psychological safety. Sample items included ‘I can speak up with recommendations/ideas for new projects or changes in procedures to my peers’ and ‘If I made a mistake on this team, I would feel safe speaking up to my peers’. The internal consistency of the full scale (α = 0.97) and individual subscales (team leader = 0.97; team members = 0.94; whole team = 0.95) were satisfactory in the current sample.

#### 2.2.5. Open-Ended Questions

A series of eight open-ended questions (Appendix A) were embedded between the above standardised scales to capture the nuances of participants’ experiences working as part of a healthcare team during the COVID-19 pandemic. These questions were designed to elicit perceived changes in how teams worked together during the pandemic, and how the participant felt about these changes.

#### 2.2.6. Covariates

A number of covariates were assessed including age (18–29, 30–39, 40–49, 50–59, 60+), sex (0 = male, 1 = female), self-reported ethnicity (0 = ethnicity other than White Irish, 1 = White Irish), length of time employed in healthcare, and whether or not the participant was redeployed due to the COVID-19 pandemic. Non-white Irish participants were grouped together for our data analysis due to the small sample size. In addition, due to small sample sizes in the 18–29 and 60+ age groups, the 18–29 and 30–39 groups were collapsed together, and the 50–59, and 60+ groups were collapsed together. This resulted in three age groups: 18–39, 40–49, 50+. These covariates were selected, following the guidelines set forth by VanderWeele [31], to ensure that the observed effects were not the result of differences among sociodemographic variables or additional work-related factors. For example, time employed in healthcare and/or being redeployed during the COVID-19 pandemic might impact an individual’s level of work engagement and/or psychological safety.

### 2.3. Data Analysis

#### 2.3.1. Quantitative Data

Structural equation modelling (SEM) was used to examine the relationships between work engagement and psychological safety, and collective leadership and organisation citizenship behaviours, while adjusting for several exogenous covariates (age, sex, self-reported ethnicity, length of time working in healthcare, and redeployment status). SEM is advantageous as it parses out measurement error, thus leading to more accurate parameter estimates [32]. It was necessary to first evaluate the fit of the measurement model (i.e., a model consisting of just the latent variables), prior to fitting the structural model [33]. Model fit was assessed using several goodness-of-fit indices [34]: Non-significant *χ*^2^, Comparative Fit Index CFI; [35] and Tucker–Lewis Index TLI; [36] values ≥ 0.90; Root Mean Square Error of Approximation RMSEA; [37] and Standardised Root-Mean-Square Residual SRMR; [38] values < 0.08 suggest adequate model fit.

Data were analysed using Mplus 8.2 [39] and the models were estimated using the robust maximum likelihood (MLR) estimator. There was a substantial proportion of missing data on certain variables of the survey. Although 100% (*n* = 152) completed the Collective Leadership Scale, 68.4% (*n* = 104) completed the UWES, 65.8% (*n* = 100) completed the Organisational Citizenship Behaviour Scale, 60.5% (*n* = 92) completed the Psychological Safety Scale, and 43.4–50% (*n* = 66–76) completed the remaining demographic questions. However, the missing data were found to be missing completely at random (MCAR), as indicated by Little’s MCAR test (*χ*^2^ [30, *n* = 152] = 18.39, *p* = 0.952).

Missing data were handled using the robust full information maximum likelihood procedure, which allows parameters to be estimated using all information available. To reduce model complexity, the latent variables (i.e., work engagement, psychological safety, collective leadership, and OCBs) were created using parcels consisting of the summed scores of each subscale within the latent variable’s respective scale. Moreover, the default procedure for using maximum likelihood estimation removes exogenous covariates using listwise deletion before the model is estimated. As such, we brought all variables, including the exogeneous covariates, into the model [39] to use all information available and thus model covariate missingness. This process makes distributional assumptions (i.e., multivariate normality) about the nature of the covariates. However, the MLR estimator was used as this estimator is robust to non-normally distributed data and can account for concerns of such multivariate non-normality.

#### 2.3.2. Qualitative Data

The eight open-ended questions included in this survey generated text responses. The qualitative data were analysed using Braun and Clarke’s [40] 6-step thematic analysis framework. This process involved repeatedly reading the data, generating initial codes and developing, refining and naming broader themes. Rather than applying a prescriptive list of codes, a bottom-up inductive approach to coding was applied which ensured themes strongly reflected the data collected. Using NVivo11 software, an experienced qualitative researcher trained in advanced qualitative design and analysis conducted line-by-line thematic coding. As themes emerged, they were deliberated and refined through discussions with the research team who were familiar with the data set. The dependability of the findings was further enhanced through deviant case analysis. By recognizing alternative viewpoints and contradicting data, a more holistic understanding of the data was achieved. Through this process, we identified patterns in the data, interpreted them, and explained their latent ideas. In total, 96 survey participants provided responses to the open-ended questions. These data provided greater insight into the experiences of teamwork during COVID-19 enhancing our understanding of the relationship between psychological safety, work engagement, collective leadership, and OCBs.

## 3. Results

### 3.1. Descriptive Statistics

Table 1 summarises the sample characteristics for the current study. The majority of participants who responded to the demographic questions are female (84%). Three quarters identified as White Irish (75%). We also note that approximately a third of our participants who responded to the demographic questions experienced redeployment to a different healthcare team during the pandemic (31.6%).

### 3.2. Measurement Model

The measurement model consisting of four latent variables (work engagement, psychological safety, collective leadership, and OCBs) demonstrated excellent statistical fit (*χ*^2^(48) = 70.46, *p* = 0.019; CFI = 0.983; TLI = 0.976; RMSEA = 0.055 [90% CI 0.023, 0.082]), SRMR = 0.035. Although a significant *χ*^2^ indicates poor model fit, this fit statistic can often reject the postulated model for trivial misspecifications [41,42]. As such, it is generally recommended to consult additional fit statistics. As the CFI, TLI, and RMSEA indicated satisfactory statistical fit, it is likely that the proposed model provided adequate fit to the data. All factor loadings were positive and significant (*p* < 0.001) ranging from 0.78–0.97 and inter-factor correlation ranged from 0.68–0.94. For individual factor loadings and inter-factor correlations see Appendix B, Table A2 and Table A3).

#### Structural Model

**Figure 1:**
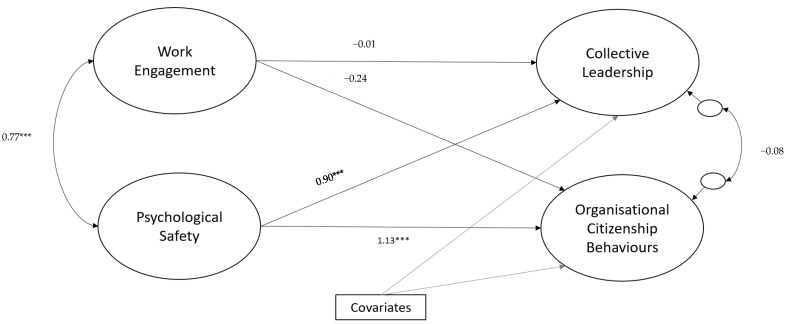
Structural model illustrating the relationship (standardised estimates) between work engagement and psychological safety, and collective leadership and organisational citizenship behaviours. Note: Individual exogenous covariate pathways are omitted for visual clarity. Statistical significance: *** *p* < 0.001.

The SEM model (see Figure 1) demonstrated satisfactory fit to the data (*χ*^2^(96) = 129.30, *p* = 0.001; CFI = 0.978; TLI = 0.969; RMSEA = 0.048 [90% CI 0.023, 0.068]), SRMR = 0.041 and explained 80.7% of the variance in collective leadership scores and 94.1% of the variance in OCBs.

While adjusting for the exogenous covariates (see Table 2 for all parameter estimates), increased psychological safety (β = 0.90, *p* < 0.001) and self-reported ethnicity (ethnicity other than White Irish) (β = −0.31, *p* < 0.001) were associated with increased collective leadership. Similarly, psychological safety (β = 1.13, *p* < 0.001) and self-reported ethnicity (ethnicity other than White Irish) (β = −0.25, *p* < 0.001) were associated with increased OCBs. Although this standardised regression coefficient (OCBs regressed on psychological safety) may appear quite large, it is important to note that standardised coefficients can exceed a value of one if there are multiple, correlated, predictors [43,44]. It is also argued that one should not modify a model for the purpose of reducing large coefficients as this can lead to biased estimates [43]. Moreover, the model converged without any indicators of improper solutions, such as negative residual variances. There was no association between work engagement and either collective leadership (β = −0.01, *p* = 0.918) or OCBs (β = −0.24, *p* = 0.055).

### 3.3. Qualitative Findings

Two key themes were generated from the inductive qualitative analysis of open-ended responses: (1) Contrasting experiences of working in a team during the pandemic; and (2) The pandemic response: a tipping point for burnout.

#### 3.3.1. Contrasting Experiences of Working in a Team during the Pandemic

Participants described greater collaboration as one of the most significant changes to occur as a result of COVID-19. Staff discussed the removal of organisational barriers and “red tape” (COV217) which commonly hindered the implementation of change. This greater autonomy resulted in enhanced innovation and implementation: “less bureaucracy and more action” (COV148). In addition to more bottom-up decision-making, participants also reported enhanced interdisciplinary teamworking characterised by improved communication and the development of “supportive networks” (COV103). Some participants described “working together as one team” rather than within discipline specific silos (e.g., medicine, nursing, allied health) (COV021), which is consistent with a shift towards a collective approach to leadership. The shared goal and challenge of responding to COVID-19 encouraged staff to “pull together” (COV 209), which increased compassion and the sense of “solidarity” (COV027) in teams. Informal ‘check-ins’ were a common support mechanism identified by participants. Many staff emphasised their desire to sustain the interprofessional teamworking that emerged during the pandemic response. Some suggested that by experiencing the benefits of collective decision-making (e.g., in service redesign), greater collaboration among team members may be sustainable. However, others questioned whether “going the extra mile” for colleagues would continue (COV104), with one participant noting that the “sense of being in this together has gone” as the pandemic continued (COV015).

Other respondents, however, described a very different experience of working in healthcare during the pandemic response. In some instances, the strictures of the traditional hierarchy and power dynamics were not only evident, but reinforced, and this was universally reported as a negative, even damaging experience for staff. Some participants outlined experiences of hierarchical decision-making in which decisions were imposed and frontline staff were expected to “follow them unquestionably like school children” (COV010). One participant emphasised the fear associated with this model of leadership: “they were working in situations of fear of the management as well as fear of the virus” (COV097). While another stressed their “frustration” at the “command and control pressures on the team” (COV094). Many staff described how this sense of powerlessness made them feel “taken for granted, not important” (COV118) and simply “just a number” (COV029). Although some participants provided examples of formal wellbeing services, others felt organisational support for frontline staff was limited or “superficial” (COV130). Some staff also described poor interpersonal relationships within their frontline team. Some explained how a “blame culture” exists (COV030), while others simply mentioned feeling “let down” (COV029). These participants suggested that COVID-19 restrictions may have strengthened the hierarchical culture that exists within their workplace. Due to social distancing precautions, some staff described how there are “less people having [a] voice at the table” (COV026). Frontline staff also recognised that personal protective equipment has impacted the informal relationships within their teams as there are “few opportunities to have chats and coffee” (COV070). For others, redeployment exacerbated the fragmented nature of teamworking: “you did what you were told without question” (COV016).

#### 3.3.2. The Pandemic Response: A Tipping Point for Burnout

Although some staff accredited greater commitment, pride and meaning to their work following their initial involvement in the pandemic response, as the pandemic continued many participants emphasised increasing levels of burnout. Burnout was illustrated in the evocative language used by participants. Staff described working on the frontline as “hell” (COV190), “unrelenting” (COV27), “exhausting, draining, and upsetting” (COV22). Some staff described feeling “shattered, shellshocked, and traumatised” (COV97) as they had “passed the novelty of being heroes” (COV143). Despite public recognition for their work, many participants felt underappreciated by their health system:

“I am working in an industry that has a small heart and little respect” (COV143).

Many participants associated burnout to their growing workloads and diminishing resources; “extra work piled on without any support” (COV005). Some described how a “get on with it” attitude exists within their organisation (COV022). However, due to the ongoing demands one participant questioned “how am I going to do this for another 20 years” (COV156). Due to their negative experiences, some staff suggested a “massive increase in anxiety related illnesses” (COV19) for healthcare staff. Others implied possibly leaving the health service because of feeling unappreciated by their organisation and the wider health system: “I’m looking for other opportunities where I’m valued more” (COV118). Despite the increased risk associated with their role (in which many contracted COVID-19), some staff felt unprotected by their health service, explaining how “no one could care less about [their] experience” (COV016). Some participants suggested that insufficiencies in organisational and wider health service leadership have left staff feeling “forgotten” on the frontline (COV019).

## 4. Discussion

This study, conducted in 2020 during the COVID-19 pandemic, aimed to capture the experiences of teamwork during the pandemic response. While previous research harnessing media and social media narratives has demonstrated that the health system can transform rapidly when presented with a single focus or threat [5], our research has used social media (Twitter) to recruit participants to a study exploring factors driving this shift towards collaborative and collective approaches to teamworking and leadership. Specifically, we hypothesised that work engagement and psychological safety would be associated with collective leadership and OCBs. Our results partially supported these hypotheses, with psychological safety, but not work engagement, predictive of collective leadership and OCBs. Qualitative analysis of text responses offered valuable contextual insight to help explain these findings.

As predicted, we found that psychological safety was associated with collective leadership behaviours. Previous research has found that the key drivers of psychological safety and its outcomes include the level of interaction between, and familiarity among, team members [45] and the quality of social relationships between team members, indicated by trust and collective thinking [46]. Where it exists, the interprofessional collaboration reported during the COVID-19 response enabled an atmosphere of psychological safety and creativity, where ideas and innovations were actively sought and developed collectively. Previous work has similarly found that inclusive approaches are associated with enhanced psychological safety and creativity [47,48]. As a result, team members were empowered to adopt leadership roles and responsibilities, effectively leveraging and contributing their expertise to support the operation and functioning of the team. Consistent with previous research [10], the qualitative findings demonstrate that the levels of peer support and the positive internal environment reported by participants promoted through this collaborative approach to change facilitated the emergence of collective leadership. This collaborative approach to change suggests a more collective mindset and coalescing around a shared goal, promoting a sense of team and collective identity [49].

In this study, psychological safety also predicted organisational citizenship behaviours. When staff feel psychologically safe in taking interpersonal risks, such as adopting a new role or responsibility, they are more willing to venture outside their own professional domain or comfort zone to support colleagues, engaging in extra-role behaviours including helping colleagues, encouraging others, and volunteering to take on additional responsibilities. However, our qualitative findings highlight the potential risk of burnout from engagement in extra-role behaviours. Organ and Ryan [50] found that individuals who engaged in high levels of OCB might feel overloaded and be at higher risk of role fatigue. Indeed, excessive levels of engagement in OCBs, such as those that were observed during the pandemic response, may contribute to burnout and ultimately disengagement in work.

Contrary to our prediction, work engagement did not predict collective leadership or OCB. Work engagement is often considered the opposite of burnout. In contrast to those who suffer from burnout, engaged employees are those who experience a sense of energetic and effective connection with their work and perceive themselves as capable of managing the demands of their role [19]. Given work engagement is defined as a positive, fulfilling work-related outlook, this experience may not be generally representative of healthcare workers perceptions during this time of unprecedented pressure on health services. Our qualitative findings offer some potential explanations into why a relationship between work engagement and collective leadership, or OCB was not observed. Firstly, this study took place several months into the onset of the global pandemic and participants in our qualitative analysis conveyed either a largely positive or a very negative experience of their work. The latter group reported higher levels of stress, burnout, and fatigue. Those who perceived more hierarchical working environments tended to report greater levels of burnout. These polarised experiences may explain why no relationship between work engagement and collective leadership and OCBs was observed. Future research should investigate this further. Secondly, whilst psychological safety and work engagement both represent positive, motivational states toward one’s work, work engagement tends to reflect cognitive appraisal of the job, whereas psychological safety is more reflective of the perceptions one holds of the work environment. This disparity may explain the current findings. Furthermore, employees tend to report high levels of work engagement when they perceive higher levels of control over their work environment [51]. In the context of an unpredictable, rapidly evolving, high-stress pandemic, it is not surprising that healthcare staff may not have the same personal resources and perceptions of control that are associated with higher levels of work engagement.

Finally, our model found that self-reported ethnicity predicted both collective leadership and OCBs. It is well-established in the literature that cross-cultural differences exist in both the meaning and perceptions of leadership and in the degree of individualism or collectivism [52]. As Friedrich et al. highlight [53], research on teams often assumes homogeneity in teams and fails to attend to this diversity. A recent meta-analysis found a positive relationship between team heterogeneity and shared leadership, suggesting the importance of diversity among team members as an antecedent condition to support collective leadership [8]. Team diversity warrants further exploration, specifically the impact of culturally diverse teams and the emergence of collective leadership and OCBs.

### 4.1. Theoretical and Practical Implications

This research delivered snapshots of healthcare workers’ experiences of teamworking during the COVID-19 pandemic to explore factors contributing to collaboration and successful initiatives during a crisis. At the point this manuscript was submitted, no peer-reviewed studies have examined the psychological safety, work engagement, collective leadership, and OCBs together in the context of healthcare, especially with the lens of teamworking during the COVID-19 pandemic. The majority of existing research in this field have examined the outcome variables separately through either a quantitative or qualitative approach, but seldom both. This study offers the evidence to support psychological safety as a precondition for collective leadership and OCBs even during a national crisis—that ‘necessity’ alone cannot force collaboration and force an environment of innovation to take place. There is considerable scope for learning how teams are adapting to the COVID-19 crisis and the factors that are promoting effective teamworking and outcomes. To date, a relatively narrow range of outcomes have been measured and there is opportunity to understand other antecedents and outcomes related to collective leadership, including aspects of workplace culture and the impact on burnout. The latter is attracting more attention recently due to the impact of the on-going COVID-19 pandemic on healthcare staff. These avenues for future research will prove fruitful in informing how we can train and develop teams to ensure the appropriate interventions to enable collective ways of working to harness intelligence and leverage skills and knowledge from the whole team to ensure optimal care delivery.

Practically, this study shows that recruitment of research participants through social media is possible, although not without its limitations. For healthcare teams, this study’s findings further underline the need to deliberately establish a psychologically safe environment, where individuals will not be humiliated for speaking up with ideas, questions, concerns, or observations. Risk-taking attitudes which are traditionally associated with innovation, requires an environment that is open to experimentation and welcoming of ideas even from the lowest ranks. Our study further shows that without such an environment, healthcare professionals are unlikely to go the extra mile. Several strategies have been identified to support healthcare teams foster psychological safety in daily practice [48,54]. Firstly, encouraging all team members to engage in more inclusive behaviours by establishing a daily multidisciplinary huddle will likely improve staff perceptions relating to the value of their role, promoting voice behaviours and staff contributions. Rather than only focusing on operational issues, protecting time monthly to reflect together as a team on more personal experiences may enhance familiarity and trust within teams. In addition to enabling team reflections, one-on-one interactions between staff have been shown to facilitate discussions on more difficult subjects [54]. Therefore, adopting an approach such as a buddy system in practice where staff are paired with a peer or more senior colleague may further strengthen interpersonal relationships promoting greater openness and ultimately psychological safety within healthcare teams.

### 4.2. Limitations

Whilst the research offers valuable insights to help us understand these rapid changes, the limitations of the work must also be acknowledged. In our attempt to be neutral, recruitment was through an academic social media channel. Even though the recruitment links were replicated over 200 times, we acknowledge that many healthcare workers without a Twitter account might not have been exposed to our study. As in all studies of this nature, we are aware that there is potentially a self-selection bias of healthcare workers who chose to participate. Those who are engaged in their work, have very positive or very negative stories to share, or feel they have the agency to make a difference are more likely to participate in this type of study.

Given the considerable burden already on healthcare staff, we deliberately adopted a design approach to minimise response burden. We designed the demographics section optional, and right at the end of the survey. Age was reported in bands, to minimise the possibility of identifying respondents from their demographics, as Ireland has a relatively small population. This resulted in participants only completing the substantive part of the survey and dropping out when they reach demographics section. The missing data observed in responses suggests the use of shortened scales through administration of only the most relevant sub-scales was warranted. Procedures to account for missing data using all information available, together with the analytical approach adopted, enabled robust analyses and inferences based on this data set and helped to ensure that this limitation was mitigated. Finally, causality cannot be inferred in cross-sectional research of this nature and the focus on staff in one national healthcare system may limit the generalisability of the findings. Future studies should use longitudinal data to capture the temporal relationship of these variables which could be moderated by prolonged pressure to healthcare staff during the COVID-19 pandemic.

## 5. Conclusions

This study explored experiences of teamworking during the COVID-19 pandemic. Structural equation modelling indicated that psychological safety, but not work engagement, was predictive of collective leadership and OCBs. Further qualitative analysis found contrasting experiences of working as part of a healthcare team during the pandemic; and initial evidence of the pandemic representing a tipping point for burnout. These findings require further investigation to clarify how the pandemic has impacted individuals and teams in the long term. Meanwhile, this research offers a valuable starting point to explore the factors driving change and the shift to more collective ways of working observed in response to demands presented as a result of the COVID-19 pandemic.

## Figures and Tables

**Table 1:** Sample characteristics and descriptive statistics of the current study.

Sample Characteristic	% (*n*)	Mean (95% CI)	Median	SD	Range
**Age**					
18–29	3.9 (3)				
30–39	23.7 (18)				
40–49	41.1 (32)				
50–59	28.9 (22)				
60+	1.3 (1)				
**Sex**					
Female	84.0 (63)				
Male	16.0 (12)				
**Self-Reported Ethnicity**					
White Irish	75.0 (57)				
Ethnicity other than White Irish	25.0 (19)				
**Redeployed**					
Yes	31.6 (24)				
No	68.4 (52)				
Work engagement		88.95 (84.52/93.39)	92.20	18.03	17–116
Psychological safety		95.61 (88.31/102.90)	108.00	29.68	19–132
Collective leadership		93.83 (86.84/100.83)	101.50	28.46	19–131
Organisational citizenship behaviours		69.88 (65.04/74.72)	74.50	19.67	14–97
Time employed in healthcare (years)		19.61 (17.39/21.83)	20.25	9.02	1.75–40.08

**Table 2:** SEM model of work engagement, psychological safety, collective leadership, and organisational citizenship behaviours.

	Collective Leadership		Organisational Citizenship Behaviours	
	B (SE)	β (SE)	B (SE)	β (SE)
**Latent variables**				
Work engagement	−0.02 (0.16)	−0.01 (0.14)	−15 (0.08)	−0.24 (0.13)
Psychological safety	0.81 *** (0.13)	0.90 (0.12)	0.53 *** (0.08)	1.13 (0.10)
**Covariates**				
Age (18–39 years)				
40–49 years	−0.10 (1.54)	−0.01 (0.10)	1.19 (0.74)	0.14 (0.09)
50 years and above	−1.44 (1.77)	−0.08 (0.11)	1.50 (0.81)	0.17 (0.09)
Sex ^a^	0.43 (1.42)	0.02 (0.07)	−0.35 (0.56)	−0.03 (0.05)
Self-reported ethnicity ^b^	−5.60 *** (1.31)	−0.31 (0.08)	−2.35 *** (0.62)	−0.25 (0.06)
Time employed in healthcare	−0.01 (0.09)	−0.01 (0.11)	−0.06 (0.04)	−0.12 (0.09)
Being redeployed	0.64 (1.15)	0.04 (0.07)	0.30 (0.62)	0.03 (0.07)

Note: B = unstandardised estimates; β = standardised estimates; SE = standard error; ^a^ = sex coded as 0 = male, 1 = female; ^b^ = self-reported ethnicity coded as 0 = ethnicity other than White Irish, 1 = White Irish. Statistical significance: *** *p* < 0.001.

## Data Availability

The data presented in this study are available on request from the corresponding author.

## Appendix A

**Table A1:** Open-Ended Questions. The following questions intend to capture attitudes towards, and motivations driving, new ways of working during COVID-19.

What, if any, is the most significant change you’ve observed in how your healthcare colleagues are working during the COVID-19 response?
Has it been a positive change or a negative change?
What has been the impact of this change?
What do you think has changed in people’s mind to enable this way of working?
Do you think this change will persist after COVID-19? What makes you say this?
Was there anything about how your team worked during COVID-19 that you felt unhappy about? Please describe.
What aspect/s of how your team worked during COVID-19 would you like to see continue?
What do you think would enable your team to sustain these ways of working?

## Appendix B

**Table A2:** Standardised factor loadings of all latent variables.

Latent Variable	β (SE)
**Factor loadings**	
**Work engagement**	
Vigour	0.96 (0.02)
Dedication	0.87 (0.05)
Absorption	0.82 (0.05)
**Psychological safety**	
Team leader	0.80 (0.05)
Team members	0.93 (0.02)
Whole team	0.92 (0.02)
**Collective leadership**	
Planning and organising	0.96 (0.01)
Problem solving	0.97 (0.01)
Support and consideration	0.92 (0.02)
**Organisational citizenship behaviours**	
Altruism	0.93 (0.02)
Courtesy	0.93 (0.03)
Civic virtue	0.78 (0.05)

Note: β = standardised estimates; SE = standard error. Statistical significance: All *p* < 0.001.

**Table A3:** Inter-factor correlations of all latent variables.

Variables	1	2	3	4
Work engagement	–			
2. Psychological safety	0.77 ***	–		
3. Collective leadership	0.68 ***	0.85 ***	–	
4. Organisational citizenship behaviours	0.68 ***	0.94 ***	0.84 ***	–

Note: Statistical significance: *** *p* < 0.001.
